# Interpretable prediction of stroke prognosis: SHAP for SVM and nomogram for logistic regression

**DOI:** 10.3389/fneur.2025.1522868

**Published:** 2025-03-04

**Authors:** Kun Guo, Bo Zhu, Lei Zha, Yuan Shao, Zhiqin Liu, Naibing Gu, Kongbo Chen

**Affiliations:** ^1^Xi’an Central Hospital, Xi’an, China; ^2^Tongchuan Mining Bureau Central Hospital, Tongchuan, China

**Keywords:** ischemic stroke, machine learning, prognosis, predictive modeling, clinical decision support

## Abstract

**Background:**

Ischemic Stroke (IS) stands as a leading cause of mortality and disability globally, with an anticipated increase in IS-related fatalities by 2030. Despite therapeutic advancements, many patients still lack effective interventions, underscoring the need for improved prognostic assessment tools. Machine Learning (ML) models have emerged as promising tools for predicting stroke prognosis, surpassing traditional methods in accuracy and speed.

**Objective:**

The aim of this study was to develop and validate ML algorithms for predicting the 6-month prognosis of patients with Acute Cerebral Infarction, using clinical data from two medical centers in China, and to assess the feasibility of implementing Explainable ML in clinical settings.

**Methods:**

A retrospective observational cohort study was conducted involving 398 patients diagnosed with Acute Cerebral Infarction from January 2023 to February 2024. The dataset included demographic information, medical histories, clinical evaluations, and laboratory results. Six ML models were constructed: Logistic Regression, Naive Bayes, Support Vector Machine (SVM), Random Forest, XGBoost, and AdaBoost. Model performance was evaluated using the Area Under the Receiver Operating Characteristic curve (AUC), sensitivity, specificity, predictive values, and F1 score, with five-fold cross-validation to ensure robustness.

**Results:**

The training set, identified key variables associated with stroke prognosis, including hypertension, diabetes, and smoking history. The SVM model demonstrated exceptional performance, with an AUC of 0.9453 on the training set and 0.9213 on the validation set. A Nomogram based on Logistic Regression was developed for visualizing prognostic risk, incorporating factors such as the National Institutes of Health Stroke Scale (NIHSS) score, Barthel Index (BI), Watanabe Drinking Test (KWST) score, Platelet Distribution Width (PDW), and others. Our models showed high predictive accuracy and stability across both datasets.

**Conclusion:**

This study presents a robust ML approach for predicting stroke prognosis, with the SVM model and Nomogram providing valuable tools for clinical decision-making. By incorporating advanced ML techniques, we enhance the precision of prognostic assessments and offer a theoretical and practical framework for clinical application.

## 1 Introduction

Ischemic stroke (IS) is a leading cause of mortality and disability worldwide, with a stark increase in global IS-related deaths reaching 3.29 million in 2023, and projections estimate a rise to 4.9 million by 2030 (1). The rapid aging and industrialization of societies, along with the spread of unhealthy lifestyle and dietary habits, have made IS the primary cause of death and disability among adults in China (2, 3). Despite advancements in the management, treatment, and prevention of IS, many patients still lack effective interventions. The prognosis of stroke is a complex process, influenced by a multitude of factors (4, 5). Timely determination of prognosis is crucial for physicians to adjust intervention strategies, prevent recurrence, ascertain adverse outcomes, and provide precision treatment plans (6). However, traditional statistical methods have limitations in terms of prognostic accuracy. The advent of machine learning has shown immense promise in handling large datasets and identifying complex patterns, offering a new horizon in the assessment of stroke prognosis.

Machine learning (ML) models have garnered attention for their prowess in handling vast datasets and discerning complex patterns (7). In the realm of prognosis assessment, these models swiftly identify independent predictive factors associated with adverse outcomes (8). The precision of these models is enhanced through a rigorous evaluation that encompasses a spectrum of metrics, including accuracy, recall, F1 score, Area Under the Receiver Operating Characteristic curve (AUC), and Shapley Additive explanations (SHAP) values. This approach not only surpasses traditional predictive methods in terms of speed but also demonstrates increasing accuracy in practical clinical applications (9).

For instance, a study optimized Principal Component Analysis (PCA) and integrated models such as Random Forest, Decision Trees, and K-Nearest Neighbors (KNN) to achieve a remarkable 98.6% accuracy rate in stroke prediction (10). Another investigation harnessed machine learning to develop a risk stratification model based on data from patients with acute ischemic stroke (AIS), which exhibited excellent predictive power as assessed by AUC values (11). Furthermore, research employing diverse machine learning algorithms to predict 90-day outcomes in stroke patients identified the Random Forest model as the ultimate predictor, with the highest AUC value.

While the performance of machine learning models is contingent upon the quality and completeness of the input data, and challenges regarding dataset representativeness and model generalizability persist, the prospects for their application in stroke prognosis assessment remain promising (12). The aim of this study is to harness clinical data to predict the 6-month prognosis of patients with cerebral infarction using machine learning algorithms and to evaluate the feasibility of explainable machine learning in clinical practice, thereby providing theoretical and practical support for its clinical application.

## 2 Materials and methods

### 2.1 Study design and participants

**Figure 1:**
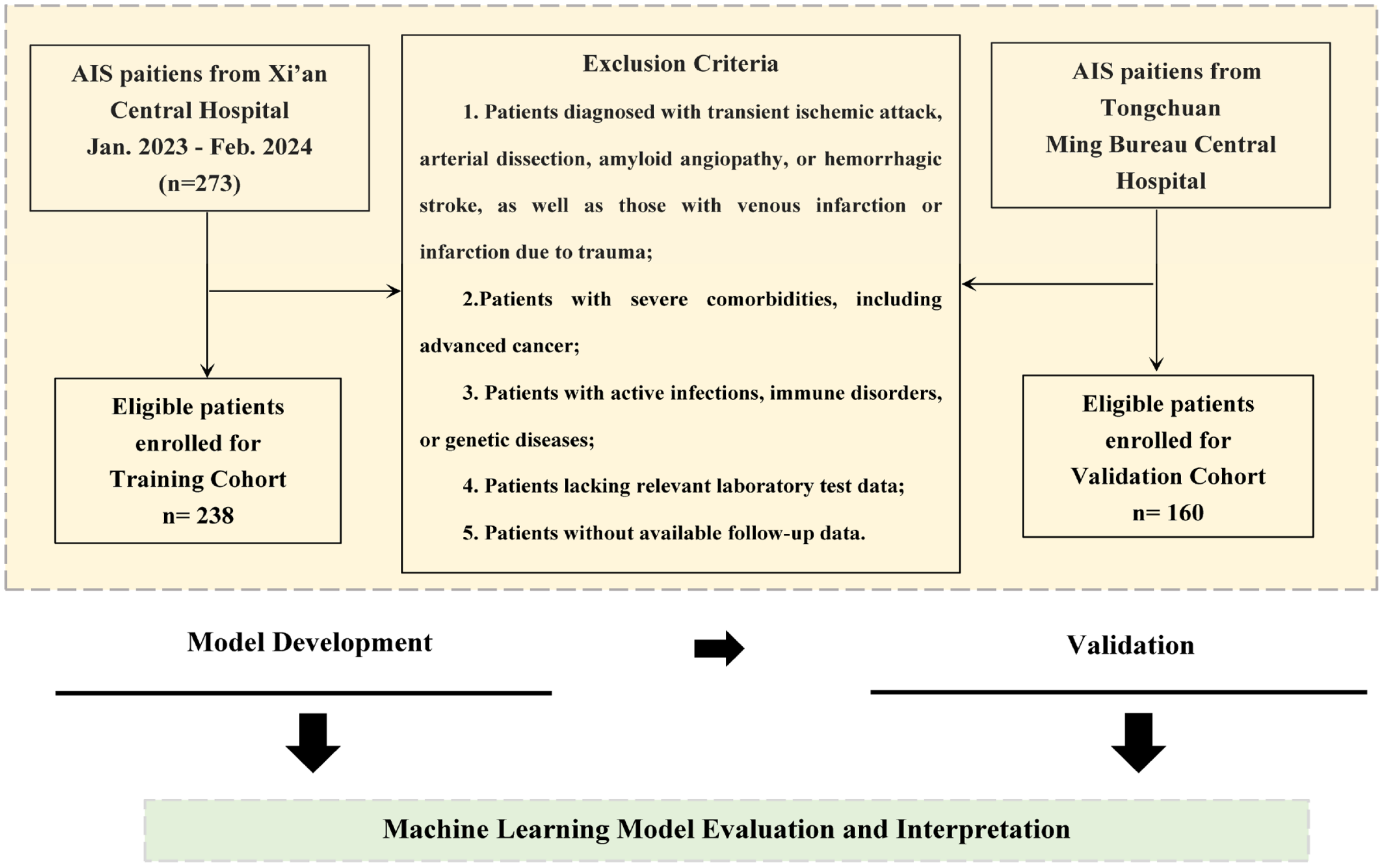
Workflow of the patient selection.

This retrospective observational cohort study included participants from two medical institutions: Xi’an Central Hospital (Center 1) and Tongchuan Mining Bureau Central Hospital (Center 2). We collected data on 474 patients diagnosed with acute cerebral infarction who visited these hospitals between January 2023 and February 2024. After applying the inclusion and exclusion criteria, 56 individuals are excluded in data loss and we ultimately retained 398 individuals to study. The administration of intravenous tissue plasminogen activator (rt-PA) conformed to the early management guidelines for acute ischemic stroke (AIS) from 2019 and 2023 (13, 14). Inclusion and Exclusion Criteria are as follows (Figure 1).

#### 2.1.1 Inclusion criteria

1. Age greater than 18 years;
2. Diagnosis of ischemic stroke within 72 h of symptom onset, in accordance with World Health Organization (WHO) criteria;
3. Received ischemic reperfusion therapy upon hospital admission.

#### 2.1.2 Exclusion criteria

1. Patients diagnosed with transient ischemic attack, arterial dissection, amyloid angiopathy, or hemorrhagic stroke, as well as those with venous infarction or infarction due to trauma;
2. Patients with severe comorbidities, including advanced cancer;
3. Patients with active infections, immune disorders, or genetic diseases;
4. Patients lacking relevant laboratory test data;
5. Patients without available follow-up data.

### 2.2 Data acquisition

In this study, a comprehensive assessment was conducted upon admission for all participants, including demographic data and medical histories with a focus on vascular risk factors and past conditions such as hypertension, hyperlipidemia, hyperuricemia, hypoalbuminemia, hyperhomocysteinemia, and type 2 diabetes mellitus. We also documented histories of smoking, alcohol consumption, atrial fibrillation, coronary heart disease, and cerebrovascular stenosis.

Clinical evaluations included the TOAST classification, blood pressure, National Institutes of Health Stroke Scale (NIHSS) score, ADL score, and the Watanabe Drinking Test. The Watanabe Drinking Test (KWST), developed by Japanese scholar Watanabe Toshio, is a method for assessing swallowing function by observing a patient’s ability to drink a specified amount of water and noting any coughing or choking. Laboratory data encompassed a wide range of tests, including complete blood count, red cell distribution width (RDW), platelet distribution width (PDW), neutrophil (NEU), lymphocyte (LYM), monocyte (MON), red blood cell (RBC) count, hemoglobin (Hb), total bilirubin (TBIL), albumin (Alb), globulin (Glb), alanine aminotransferase (ALT), aspartate aminotransferase (AST), urea, creatinine (Cr), uric acid (UA), total cholesterol (TC), triglycerides (TG), apolipoprotein A-I (ApoAI), high-density lipoprotein (HDL), low-density lipoprotein (LDL), glucose (Glu), and homocysteine (HCY). Electrolyte levels (K, Na, Cl, Ca, P) and coagulation functions (PT, INR, APTT, TT, FIB, DD, FDP) were assessed. Cardiac function was evaluated with ejection fraction (EF) and heart rate (HR), and nutritional status with the Geriatric Nutritional Risk Index (GNRI) (15–19).


SII=α×lymphocytes+β×monocytes+γ×neutrophils

$$PLR=\frac{\text{plateltes}}{\text{lymphocytes}}$$

$$MLR=\frac{\text{monocytes}}{\text{lymphocytes}}$$

$$NLR=\frac{\text{neutrophils}}{\text{lymphocytes}}$$

$$\text{CONUT}=\text{albumin}+\frac{\text{cholesterol}}{10}+\frac{\text{total}\;\text{lymphocyte}\;\text{count}}{10}$$

These indices, derived from routine blood tests, offer clinicians a swift and effective tool for assessing a patient’s inflammatory status. When these ratios are elevated, they are frequently indicative of heightened inflammatory activity, which can be instrumental in diagnosing and monitoring of a variety of inflammation conditions.

### 2.3 Prognosis assessment

Prognosis assessment was conducted by two licensed neurologists who were trained in the standardized mRS (modified Rankin Scale) scoring system and were blinded to the study’s objectives. They independently evaluated the patients’ outcomes through telephone interviews at the 3-month mark following discharge. In instances where there was a disagreement between the two neurologists, a third senior neurologist, also blinded to the study, was consulted to make the final determination. The prognosis was dichotomized into two categories: good prognosis, defined as an mRS score of 2 or less, and poor prognosis, indicated by an mRS score greater than 2.

### 2.4 Feature selection

In this study, we evaluated the prognostic predictive accuracy of various clinical indicators for stroke patients using Receiver Operating Characteristic (ROC) curves and Area Under the Curve (AUC) values. We employed heatmaps to visually represent the correlation coefficients among different clinical indicators, thereby elucidating their interrelationships. To reduce the risk of multicollinearity, we initially excluded features with Spearman correlation coefficients greater than 0.9. Following this, we utilized the LASSO (Least Absolute Shrinkage and Selection Operator) regression model to further refine our variable selection. Cross-validation was employed to determine the optimal regularization parameter, Lambda, which is essential for balancing model complexity and predictive performance. By using the “lassoCV” function, we assessed the mean squared error (MSE) across a spectrum of Lambda values to pinpoint the value that minimizes MSE, thereby identifying the optimal Lambda. Our five-fold cross-validation analysis revealed that an optimal log Lambda value of approximately-4 provided the best model discrimination and predictive accuracy. After applying LASSO and subsequent multivariate analysis, we selected the variables to be included in the model.

### 2.5 Model development and performance comparison

We then proceeded to construct six distinct machine learning models to forecast the mRS score at the 6-month post-discharge mark for patients suffering from acute ischemic stroke. The models included logistic regression, naive Bayes, support vector machine (SVM), random forest, XGBoost, and AdaBoost. Each model’s performance was rigorously evaluated using a suite of metrics: the area under the receiver operating characteristic curve (AUC), sensitivity, specificity, predictive values, and the F1 score. To ascertain the robustness and generalizability of our models, a five-fold cross-validation approach was employed. This technique involves partitioning the data into five segments to ensure a comprehensive assessment.

### 2.6 Interpretability of machine learning models

The interpretability of machine learning models is pivotal for elucidating the predictions made by ML models and for quantifying the influence of individual features on these predictions. To enhance the interpretability of our logistic regression model, we employed a Nomogram. A Nomogram is an intuitive graphical tool that consolidates the effects of multiple predictive variables into a single visual representation, making it an excellent choice for improving model transparency and interpretability. It visually represents the contribution of each variable to the prediction outcome through scaled line segments, which significantly boosts the model’s clarity and interpretability. Furthermore, we utilized the SHAP (SHapley Additive exPlanations) method to interpret the SVM model. The SHAP method enables interpretation of model predictions at both the individual patient level and the cohort level. By computing the SHAP values for each feature across all patients and averaging them, we can gauge the significance of each feature in predicting outcomes. The SHAP feature importance plot unveils the global impact of features, where a higher average absolute SHAP value indicates a more substantial contribution to the model’s predictions. The SHAP summary plot further delineates the specific influence of each feature on model predictions, with each plot point representing the SHAP value of a particular feature for an individual patient, and the color gradient from red to blue indicating the magnitude of the feature values. Additionally, SHAP dependency plots provide insights into how changes in specific feature values affect model predictions. These analyses were conducted using SHAP version 0.46.0 within a Python environment.

### 2.7 Statistical analysis

Statistical analyses in this study were conducted using Python 3.12.1 and R 4.4.1. Data normality was assessed with the Kolmogorov–Smirnov test. For normally distributed continuous variables, we used independent samples *t*-tests; for non-normally distributed data, Mann–Whitney U tests were applied. Categorical variables were analyzed with chi-square tests or Fisher’s exact tests when appropriate. Machine learning models were also constructed for prediction. We considered *p* < 0.05 (two-tailed) as statistically significant.

## 3 Results

### 3.1 Baseline data

Center 1 (*n* = 238) served as the training set for model development, while Center 2 (*n* = 160) functioned as the internal validation set. In the training set, univariate analysis identified significant differences in several key variables between the good and poor prognosis groups: hypertension (*p* < 0.001), diabetes (*p* < 0.001), smoking history (*p* < 0.001), alcohol consumption history (*p* = 0.043), platelet distribution width (PDW) (*p* < 0.001), hyperhomocysteinemia (*p* = 0.002), apolipoprotein A1 (ApoAI) (*p* = 0.014), activated partial thromboplastin time (APTT) (*p* = 0.006), D-dimer (DD) (*p* = 0.03), Barthel Index (BI) (*p* < 0.001), and the National Institutes of Health Stroke Scale (NIHSS) (*p* < 0.001). These variables could be linked to disease occurrence, progression, or prognosis. Variables such as age, gender, hyperlipidemia, hyperuricemia, hypoalbuminemia, white blood cell count, red blood cell count, hemoglobin, hematocrit, platelet count, albumin, globulin, etc., did not show significant differences (*p* ≥ 0.05), suggesting they may not be the primary drivers of prognosis (Table 1).

**Table 1:** Baseline characteristics of patients in training and validation cohorts.

Variable	Training cohort *n* = 238		p.overall	Validation cohort n = 160		p.overall
	mRS ≤ 2 *n* = 75	mRS>2 *n* = 163		mRS ≤ 2 *n* = 53	mRS>2 *n* = 107	
Age	68.6 (11.7)	66.6 (11.4)	0.219	67.7 (12.9)	68.3 (11.7)	0.762
Gender	0.79 (0.41)	0.77 (0.42)	0.814	0.70 (0.46)	0.75 (0.44)	0.518
Hypertension	0.53 (0.50)	0.78 (0.42)	<0.001	0.53 (0.50)	0.76 (0.43)	0.004
Hyperlipemia	0.23 (0.42)	0.34 (0.48)	0.058	0.25 (0.43)	0.34 (0.47)	0.228
Hyperuricemia	0.08 (0.27)	0.15 (0.36)	0.112	0.17 (0.38)	0.19 (0.39)	0.791
Hypoproteinemia	0.13 (0.34)	0.12 (0.32)	0.721	0.21 (0.41)	0.12 (0.33)	0.186
Hyperhomocysteinemia	0.43 (0.50)	0.64 (0.48)	0.002	0.55 (0.50)	0.65 (0.48)	0.201
Diabetes	0.21 (0.41)	0.58 (0.49)	<0.001	0.30 (0.46)	0.60 (0.49)	<0.001
SmokingHistory	0.11 (0.31)	0.65 (0.48)	<0.001	0.23 (0.42)	0.63 (0.49)	<0.001
DrinkingHistory	0.05 (0.23)	0.13 (0.34)	0.043	0.06 (0.23)	0.14 (0.35)	0.074
Stroke	0.29 (0.46)	0.45 (0.62)	0.027	0.34 (0.48)	0.42 (0.51)	0.328
AF	0.13 (0.34)	0.06 (0.23)	0.075	0.06 (0.23)	0.07 (0.26)	0.658
AS	0.51 (0.50)	0.73 (0.45)	0.001	0.34 (0.48)	0.76 (0.43)	<0.001
Hemadostenosis	0.51 (0.50)	0.58 (0.49)	0.277	0.25 (0.43)	0.56 (0.50)	<0.001
WBC	7.11 [5.49;8.66]	6.86 [5.68;9.23]	0.659	7.14 [6.05;9.43]	6.71 [5.30;8.09]	0.076
RDW	13.2 [11.5;15.5]	13.5 [12.3;15.6]	0.152	12.9 [11.6;15.5]	14.4 [12.8;16.4]	0.108
PDW	16.9 [16.2;19.3]	19.4 [16.5;22.1]	<0.001	18.3 [16.4;19.5]	19.7 [16.6;22.7]	0.006
NEU	5.89 [3.66;6.86]	4.99 [3.69;6.70]	0.494	5.22 [4.25;7.57]	4.74 [3.44;6.12]	0.048
MON	0.47 [0.37;0.64]	0.46 [0.35;0.61]	0.616	0.44 [0.36;0.56]	0.44 [0.35;0.63]	0.9
RBC	4.57 [4.28;4.88]	4.52 [4.22;5.04]	0.6	4.65 [4.31;4.93]	4.55 [4.22;4.85]	0.294
Hb	144 [136;153]	144 [131;154]	0.598	143 [134;153]	143 [130;152]	0.433
HCT	0.44 [0.41;0.47]	0.44 [0.40;2.38]	0.119	0.44 [0.40;0.48]	0.44 [0.40;1.31]	0.808
PLT	184 [140;220]	176 [142;222]	0.935	180 [156;216]	182 [141;213]	0.477
TBIL	18.5 [14.8;23.7]	15.7 [12.4;21.5]	0.039	17.2 [14.0;23.7]	17.4 [13.1;23.0]	0.526
Alb	38.8 [36.2;41.9]	39.1 [36.7;41.8]	0.626	39.6 [35.7;42.7]	39.9 [37.0;42.2]	0.646
Glb	21.5 [19.7;24.4]	22.8 [20.0;27.1]	0.104	22.6 [19.3;27.8]	22.5 [20.1;27.0]	0.793
ALT	17.0 [12.0;21.5]	18.0 [12.0;24.0]	0.613	17.0 [12.0;22.0]	16.0 [11.0;21.5]	0.986
AST	20.0 [17.0;23.5]	19.0 [16.0;25.0]	0.493	18.0 [16.0;23.0]	18.0 [16.0;23.0]	0.863
AST.ALT	1.89 [1.00;2.50]	1.56 [1.09;2.38]	0.601	1.80 [1.06;2.38]	1.69 [1.08;2.16]	0.663
Urea	5.87 [4.52;6.83]	5.27 [4.61;6.62]	0.177	5.31 [5.06;5.85]	5.34 [4.36;6.10]	0.66
Cr	65.0 [56.2;74.9]	66.0 [56.6;76.0]	0.363	68.5 [57.4;78.0]	65.8 [56.3;76.1]	0.41
UA	301 [244;361]	318 [263;384]	0.119	321 [272;393]	315 [270;374]	0.666
TC	3.89 [3.39;4.66]	4.03 [3.50;4.88]	0.267	4.41 (1.14)	4.43 (1.29)	0.908
TG	1.26 [0.90;1.71]	1.36 [0.96;1.90]	0.209	1.15 [0.76;1.50]	1.33 [0.99;1.94]	0.022
ApoAI	1.21 [1.07;1.46]	1.32 [1.12;1.88]	0.014	1.32 [1.10;1.94]	1.32 [1.15;1.67]	0.997
HDL	1.10 [0.97;1.21]	1.04 [0.91;1.27]	0.671	1.16 [0.99;1.32]	1.09 [0.94;1.31]	0.469
LYM	1.56 [1.12;1.89]	1.67 [1.27;2.05]	0.376	1.54 [1.05;2.19]	1.63 [1.25;2.20]	0.351
LDL	2.14 [1.75;2.78]	2.34 [1.82;2.92]	0.152	2.56 [1.85;3.06]	2.42 [1.70;3.20]	0.649
Glu	6.08 [5.16;7.29]	5.71 [5.04;8.09]	0.563	6.74 [5.33;7.86]	5.87 [5.18;7.44]	0.154
HCY	19.5 [14.1;29.5]	18.8 [14.0;25.4]	0.39	20.0 [14.2;31.8]	18.3 [13.2;29.3]	0.463
K	4.08 [3.80;4.30]	4.07 [3.80;4.34]	0.805	3.90 [3.62;4.30]	4.09 [3.80;4.30]	0.305

Signif. codes: 0 “***” 0.001 “**” 0.01 “*” 0.05 “.” 0.1 “” 1.

### 3.2 Univariate ROC and AUC analysis

In our study, we assessed the prognostic accuracy of various clinical indicators for stroke patients using Receiver Operating Characteristic (ROC) curves (Supplementary Figure S1A) and Area Under the Curve (AUC) values (Supplementary Figure S1B). The analysis revealed several key indicators with high sensitivity, including hypertension, diabetes, smoking history, aortic stenosis, blood viscosity, neutrophil proportion, platelet distribution width (PDW), aspartate aminotransferase, activated partial thromboplastin time (APTT), D-dimer, systemic immune-inflammation index (SII), platelet-to-lymphocyte ratio (PLR), Barthel Index (BI), and National Institutes of Health Stroke Scale (NIHSS). Notably, BI and NIHSS exhibited exceptionally high sensitivity values, with AUCs of 0.98 and 1.00, respectively, highlighting their critical role in assessing post-stroke functional prognosis. APTT, D-dimer, and SII also showed high sensitivity with AUC values of 0.98, 1.00, and 0.75, respectively, likely due to their importance in monitoring coagulation and inflammation in stroke patients. The AUC for NIHSS was 0.84, indicating strong discriminative power. Other indicators such as smoking history, PDW, blood viscosity, hyperhomocysteinemia, and diabetes demonstrated significant discriminative power with AUC values ranging from 0.68 to 0.76. In contrast, traditional cardiovascular risk factors and demographic indicators like age, gender, and heart rate had lower AUC values, suggesting their limited predictive utility in stroke prognosis. In conclusion, our AUC analysis identified a set of clinical indicators with robust discriminative power for stroke prognosis, particularly NIHSS, smoking history, and PDW, which can serve as valuable tools in evaluating stroke outcomes.

### 3.3 Correlation analysis using heatmaps

We employed heatmaps to graphically display the correlation coefficients among various clinical indicators, providing a clear visual representation of their relationships. Notable findings include an exact correlation coefficient of 1 between Smoking History and Drinking History, signifying an extremely significant positive correlation. A strong negative correlation was identified between APTT and DD, with a correlation coefficient of −0.62, indicating that as one increases, the other tends to decrease significantly. Conversely, a strong positive correlation was observed between FDP and DD, with a coefficient of 0.98, suggesting a high degree of direct relationship. The correlation coefficient of −0.64 between BI and NIHSS underscores the relationship between post-stroke functional prognosis and stroke severity. Hyperhomocysteinemia, TBIL, and SII exhibited moderate correlations with several variables, with correlation coefficients of −0.2, −0.2, and −0.4, respectively, indicating a moderate inverse relationship (Supplementary Figure S2).

### 3.4 Feature selection process

**Figure 2:**
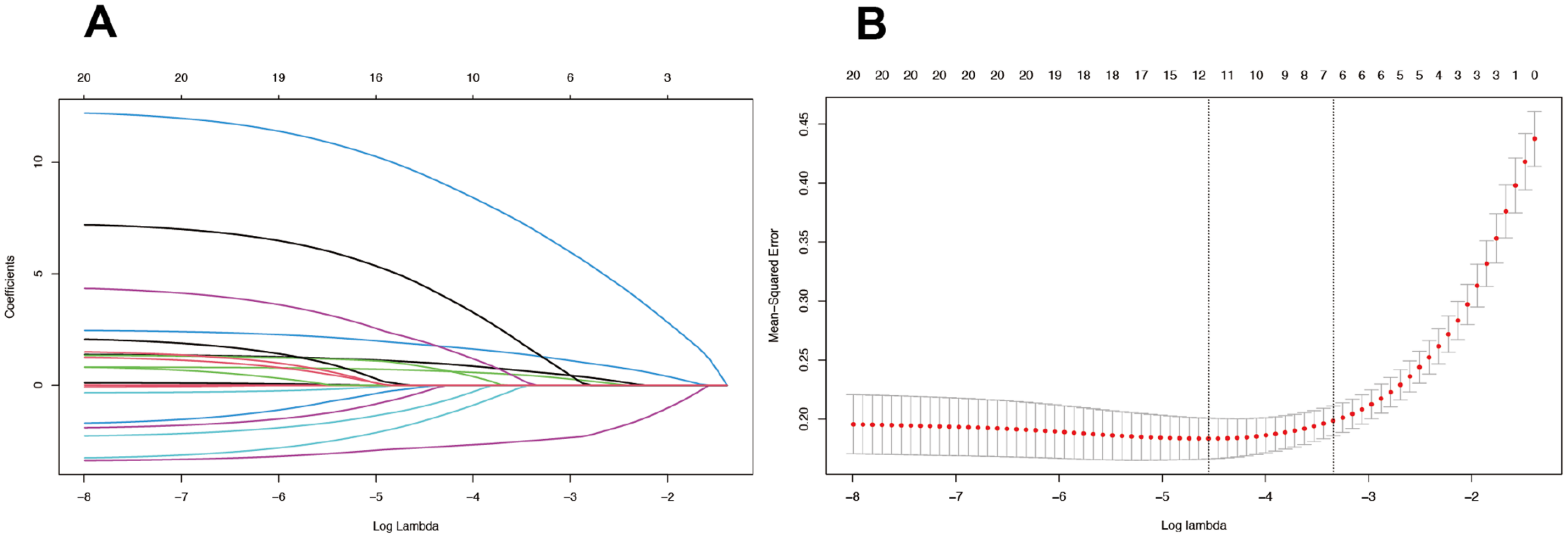
The LASSO model, employing a tuning parameter (λ) and utilizing fivefold cross-validation with both minimum and 1se criteria **(B)**, was used to select radiomics features during the feature selection process **(A)**.

In our study, the feature selection process initially excluded features with Spearman correlation coefficients exceeding 0.9 to mitigate the risk of multicollinearity. Subsequently, we employed the LASSO regression model (Figure 2) to further refine our variable selection, and based on this analysis, we selected nine variables of significant importance for predicting the prognosis of stroke patients: National Institutes of Health Stroke Scale (NIHSS), Barthel Index (BI), Watanabe Drinking Test (KWST), Apolipoprotein A1 (ApoAI), Platelet Distribution Width (PDW), Aortic Stenosis (AS), Smoking History, Diabetes, and Hypertension. Following subsequent multivariate analysis, (Table 2) we further identified several significant factors that influence stroke prognosis, including hypertension, diabetes, smoking history, Platelet Distribution Width (PDW), Watanabe Drinking Test (KWST) score, Barthel Index (BI), and National Institutes of Health Stroke Scale (NIHSS). These variables were then integrated into a nomogram model, serving as a predictive tool for poor prognosis in stroke patients.

**Table 2:** Logistic regression analysis results.

Variable	OR	95% CI lower	95% CI upper	Estimate	Std. Error	*z*-Value	*p*-Value	Significance
Hypertension	2.675	1.104	6.76	0.984	0.459	2.145	0.032	*
Diabetes	3.612	1.475	9.353	1.284	0.468	2.744	0.006	**
SmokingHistory	12.98	4.881	39.738	2.563	0.53	4.84	<0.001	***
Atherosclerosis	2.461	0.974	6.431	0.901	0.478	1.884	0.06	.
Platelet distribution width	1.262	1.057	1.544	0.232	0.096	2.424	0.015	*
Apolipoprotein AI	1.004	0.999	1.009	0.004	0.002	1.737	0.082	.
KWST	0.411	0.178	0.812	−0.889	0.379	−2.344	0.019	*
Barthel index	0.953	0.917	0.988	−0.048	0.019	−2.533	0.011	*
NIHSS	1.345	1.05	1.76	0.297	0.13	2.289	0.022	*

Signif. codes: 0 “***” 0.001 “**” 0.01 “*” 0.05 “.” 0.1 “” 1.

### 3.5 Model development and performance comparison

After comparative analysis, we have compiled a performance comparison of six machine learning models (Table 3). This clearly presents the performance of each model across various evaluation metrics, providing a straightforward basis for assessing their strengths and weaknesses. These data are valuable for understanding the performance of different models in medical prediction tasks and for guiding future research and clinical practice.

**Table 3:** Predictive performance of machine learning model in training and validation cohorts.

Model name	Accuracy	AUC	95% CI	Sensitivity	Specificity	PPV	NPV	Precision	Recall	F1	Threshold	Task
LR	0.861345	0.933824	0.9017–0.9659	0.852761	0.88	0.939189	0.733333	0.939189	0.852761	0.893891	0.663912	Label-train
LR	0.825	0.903192	0.8569–0.9494	0.831776	0.811321	0.89899	0.704918	0.89899	0.831776	0.864078	0.730308	Label-test
NaiveBayes	0.865546	0.934888	0.9040–0.9658	0.846626	0.906667	0.951724	0.731183	0.951724	0.846626	0.896104	0.765105	Label-train
NaiveBayes	0.7875	0.916064	0.8754–0.9567	0.682243	1	1	0.609195	1	0.682243	0.811111	0.978566	Label-test
SVM	0.894958	0.945276	0.9172–0.9734	0.907975	0.866667	0.936709	0.8125	0.936709	0.907975	0.922118	0.704345	Label-train
SVM	0.79375	0.921354	0.8820–0.9607	0.700935	0.981132	0.986842	0.619048	0.986842	0.700935	0.819672	0.889543	Label-test
RandomForest	0.970588	0.995992	0.9914–1.0000	0.957055	1	1	0.914634	1	0.957055	0.978056	0.8	Label-train
RandomForest	0.8	0.835302	0.7649–0.9057	0.831776	0.829787	0.864078	0.684211	0.864078	0.831776	0.847619	0.8	Label-test
XGBoost	0.953782	0.992147	0.9855–0.9988	0.96319	0.933333	0.969136	0.921053	0.969136	0.96319	0.966154	0.589629	Label-train
XGBoost	0.80625	0.867307	0.8109–0.9237	0.766355	0.886792	0.931818	0.652778	0.931818	0.766355	0.841026	0.802483	Label-test
AdaBoost	0.92437	0.973947	0.9583–0.9896	0.944785	0.88	0.944785	0.88	0.944785	0.944785	0.944785	0.509112	Label-train
AdaBoost	0.7375	0.870129	0.8143–0.9259	0.635514	0.943396	0.957746	0.561798	0.957746	0.635514	0.764045	0.583166	Label-test

### 3.6 Explanation of the nomogram model

**Figure 3:**
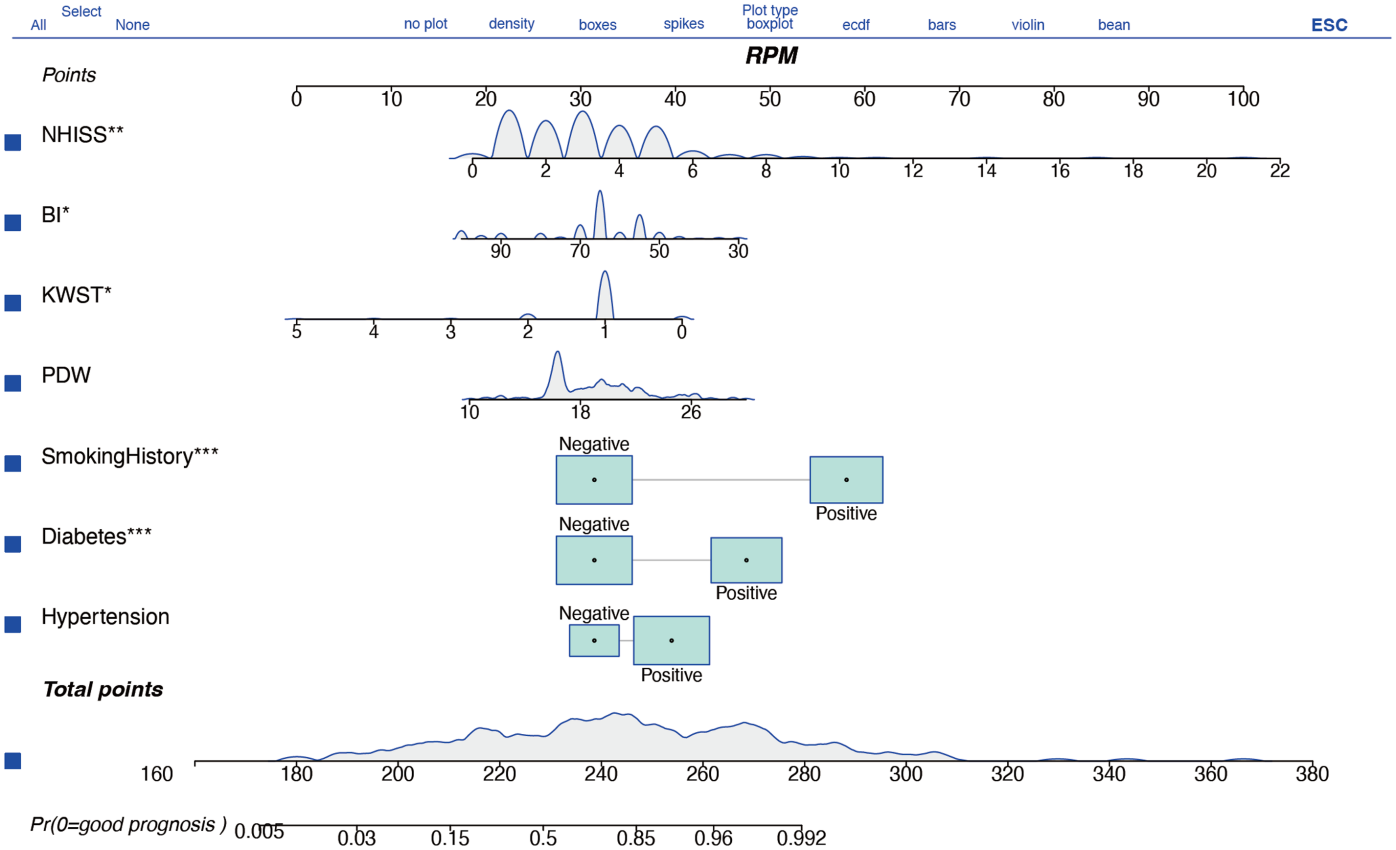
A nomogram based on logistic regression for clinical decision-making.

The nomogram model is an intuitive tool for evaluating the prognostic risk of individual stroke patients, assigning points to various factors based on their impact on the likelihood of a poor outcome. Key predictors such as hypertension, diabetes, and particularly smoking history, are identified as having significant influence, with smoking history being the most impactful. An increased platelet distribution width (PDW) is associated with a higher risk of poor prognosis, and continuous variables like the Watanabe Drinking Test (KWST) score, Barthel Index (BI), and National Institutes of Health Stroke Scale (NIHSS) also contribute positively to the risk assessment, with higher scores indicating a greater risk. The total score on the nomogram, ranging from 260 to 480 points, reflects the overall risk, with higher scores suggesting a higher probability of a poor prognosis. For instance, a score of 380 points might correspond to an approximately 0.8 probability of a poor prognosis, as read from the “Pr (+)” line on the nomogram, where Pr =1 represents a poor prognosis and Pr =0 a good one (Figure 3).

#### 3.6.1 ROC analysis

**Figure 4:**
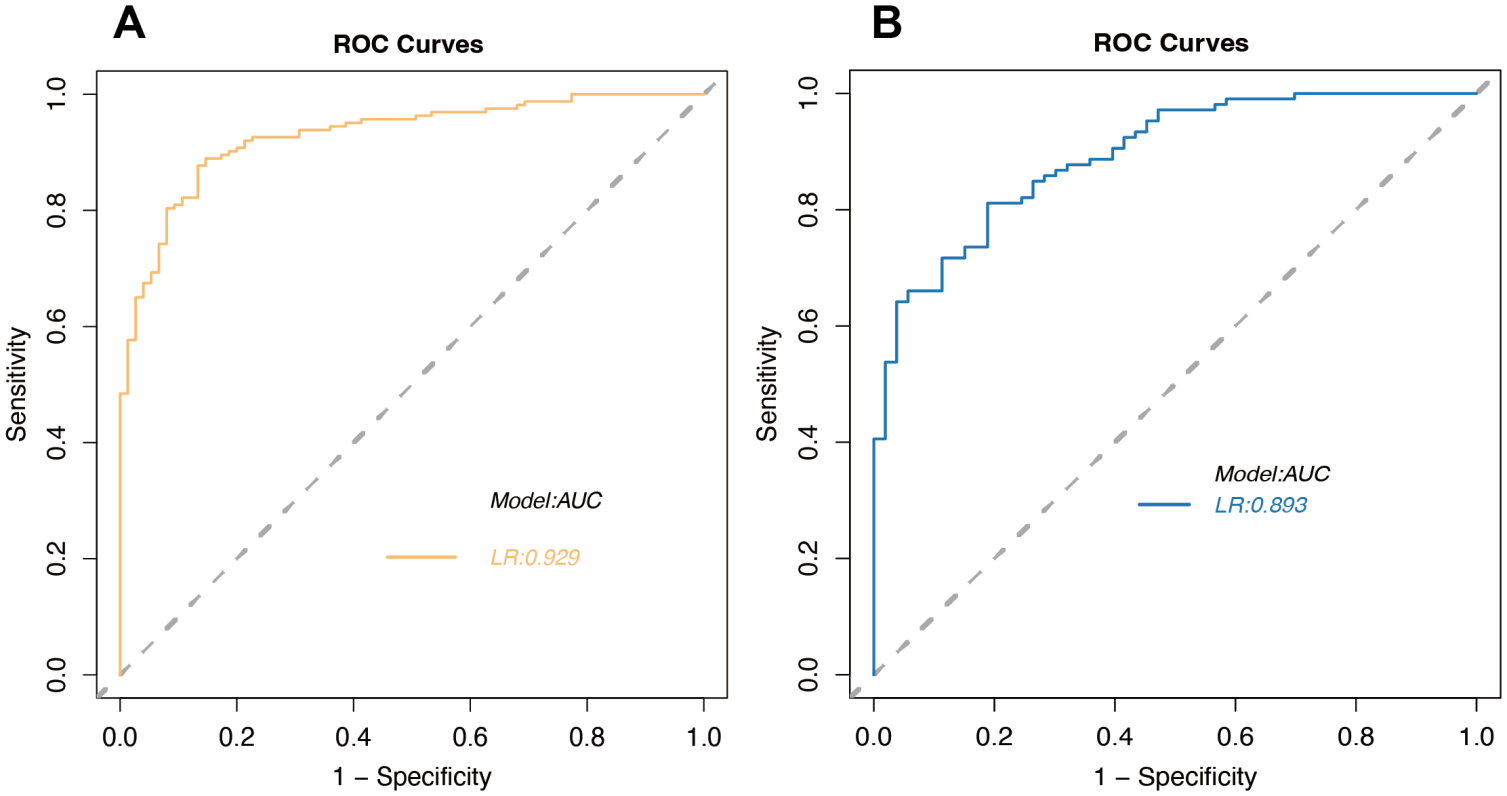
Receiver Operating Characteristic (ROC) curves for logistic regression in the training set **(A)** and validation set **(B)**.

In our study, we performed a thorough ROC analysis to assess the prognostic accuracy for stroke patients. The training set’s ROC analysis yielded an AUC of 0.929, showcasing the model’s superior ability to differentiate between patients with favorable and unfavorable outcomes (Figure 4A). An AUC approaching 1 indicates exceptional model accuracy, which is crucial for informed clinical decision-making. The validation set’s AUC was 0.893, slightly lower than the training set, yet still indicative of the model’s robust discrimination ability (Figure 4B). This suggests that the model retains high predictive accuracy on new, unseen data, highlighting its strong generalization potential.

#### 3.6.2 Calibration curve analysis

**Figure 5:**
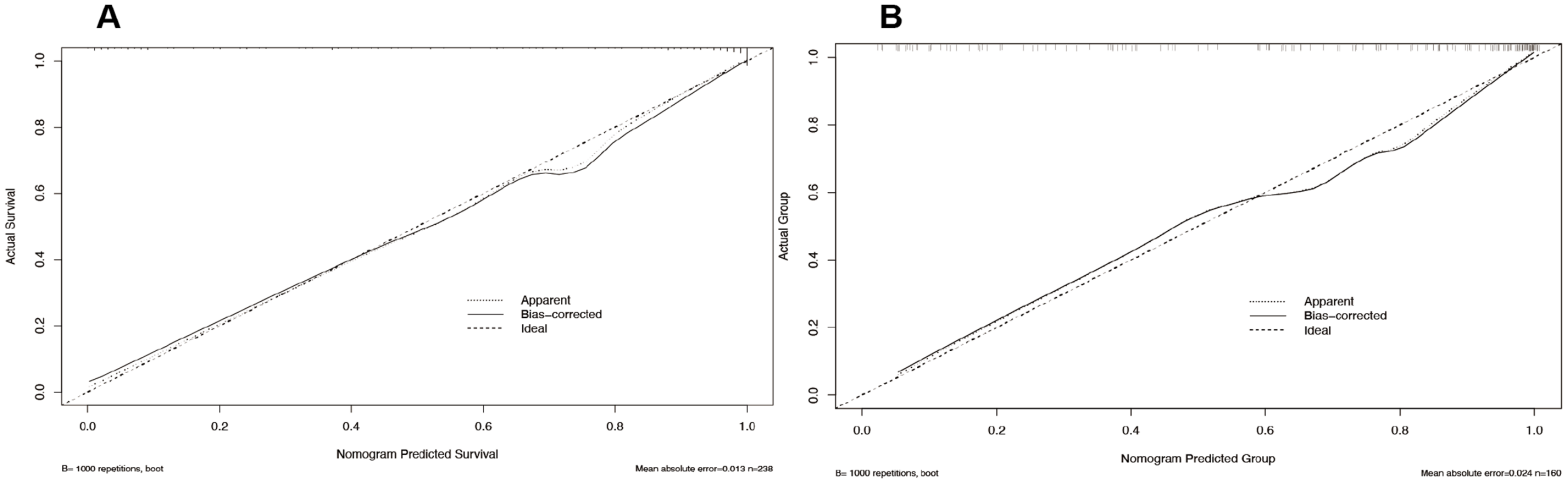
Calibration curves for logistic regression: training set **(A)** and validation set **(B)**.

In our study, we rigorously evaluated the predictive accuracy of our model by comparing the predicted probabilities against actual outcomes using calibration curve analysis. The calibration curve for the training set closely aligned with the diagonal line, signifying a high level of concordance between the model’s predictions and the actual survival probabilities (Figure 5A). This alignment, coupled with a mean absolute error (MAE) of 0.013, underscored the model’s exceptional predictive precision. To bolster the model’s reliability, we subjected it to 1,000 bootstrap repetitions for refinement, thereby further enhancing the stability and accuracy of its predictions. For the validation set, the calibration curve closely adhered to the diagonal line, indicating that the model preserved predictive consistency when applied to new data. The mean absolute error (MAE) for the validation set was 0.024, which, while slightly higher than that of the training set, remained within an acceptable range (Figure 5B). This suggests that the model retains high predictive accuracy on unseen data, a crucial measure of its generalization capability. To address any potential bias, multiple bootstrap repetitions were employed, further solidifying the robustness of the model’s predictive performance. Synthesizing the analysis from both the training and validation sets, our model exhibited commendable predictive accuracy and stability across both, reinforcing its reliability in practical application.

#### 3.6.3 Decision curve analysis

In this study, we employed Decision Curve Analysis (DCA) to evaluate the clinical utility of our machine learning model in predicting cerebral infarction. DCA is a method used to assess the clinical benefit of predictive models by comparing the net benefit between model predictions and actual clinical decisions, thereby helping to determine the clinical value of the model at different risk thresholds. The net benefit reflects the relative advantage of using the model for decision-making compared to not using it at a specific risk threshold. In the training set, the decision curve analysis revealed that the net benefit of using the model surpassed that of not using it at a risk threshold of approximately 0.2, with further increases in net benefit as the threshold rose. This indicates that the model provides greater benefits for clinical decision-making at higher risk thresholds. Non-parametric statistical validation using the Mann–Whitney U test confirmed that the decision curve for the training set was significantly different from the “no model” approach at risk thresholds above 0.2 (*p* < 0.05), further substantiating the model’s clinical superiority at these thresholds.

**Figure 6:**
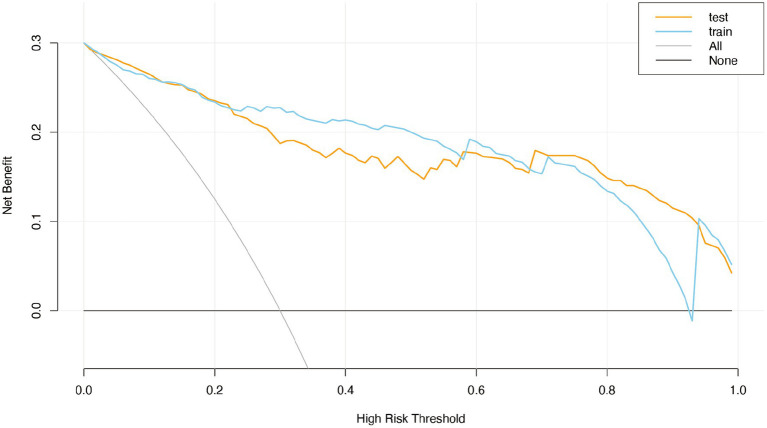
The clinical decision curve (DCA) of the logistic regression model for the training set and the validation set.

In the validation set, the decision curve mirrored the results of the training set, demonstrating the model’s robust generalization ability and significant deviation from the “no model” strategy at risk thresholds above 0.2 (*p* < 0.05). This consistency indicates that the model can provide reliable clinical benefits on independent datasets. Combining the analyses of both the training and validation sets, DCA further confirmed the model’s clinical utility across various risk thresholds, with the net benefit of the model significantly exceeding that of the “no model” strategy at thresholds above 0.2 (*p* < 0.05). These results suggest that our machine learning model not only achieves high accuracy in predicting cerebral infarction but also provides valuable decision support for clinicians in real-world applications, thereby improving patient outcomes. In conclusion, the DCA findings emphasize the significant clinical value of our machine learning model in predicting cerebral infarction. The model’s superior net benefit above a specific risk threshold indicates its important potential for application in clinical decision-making processes (Figure 6).

### 3.7 Explanation of SHAP condensed waterfall plot

**Figure 7:**
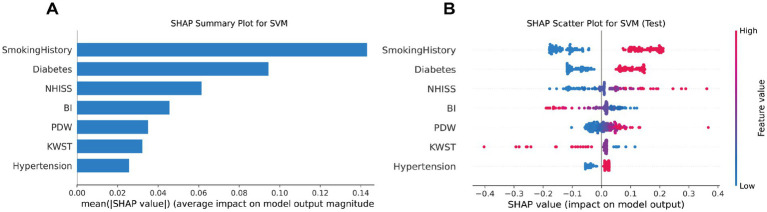
SVM model SHAP value summary plot **(A)**, and SVM model SHAP value scatter plot **(B)**.

SHAP condensed waterfall plots visually depict feature contributions to machine learning predictions. Arrows represent each feature’s effect, with longer arrows indicating greater influence. Red arrows signify positive impacts on predictions, while blue ones denote negative impacts. Starting from the average predicted value, arrows illustrate feature-induced changes to the initial forecast. The final prediction is the aggregate of these changes. In our SVM model analysis, SHAP plots highlighted NIHSS, PDW, and BI as the most influential predictors for stroke prognosis, with higher SHAP values than other variables. Despite being less impactful, factors like hypertension, KWST, diabetes, and smoking history also played a role. This emphasizes the significance of monitoring NIHSS, PDW, and BI for accurate stroke prognosis assessments (Figure 7A).

#### 3.7.1 Interpreting the SHAP scatter plot

The SHAP scatter plot uses arrows to show the effect of each feature on model predictions. Longer arrows indicate a greater impact, with red arrows pointing to an increase and blue arrows to a decrease in predicted probabilities. The plot starts from the average prediction, and the arrows show how each feature modifies this baseline. The SHAP scatter plot for our SVM model highlights that NIHSS, PDW, and BI are the most influential features in predicting stroke prognosis, with higher SHAP values than other variables. Despite being less impactful, factors like hypertension, KWST, diabetes, and smoking history also play a role. This emphasizes the significance of monitoring NIHSS, PDW, and BI for accurate stroke prognosis (Figure 7B).

SHAP analysis highlights the key clinical factors influencing stroke risk. Diabetes and smoking history show significant SHAP value changes, indicating their crucial roles. PDW and KWST also have notable impacts. BI’s interaction with smoking history suggests a complex link between smoking and functional impairment. These insights can inform clinical prevention strategies for stroke (Supplementary Figure S3).

#### 3.7.2 Interpreting the SHAP force plots for two patients

**Figure 8:**
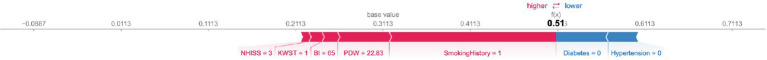
SHAP force plot showing feature contributions.

By juxtaposing the SHAP condensed waterfall plots of these two patients, we can visually discern the disparate impacts of various features on their respective stroke predictions. This visual comparison elucidates how the model incorporates different features to make predictions and identifies which features are pivotal in determining the risk of stroke. Patient NO.45 has a 51% probability of poor prognosis (Figure 8).

## 4 Discussion

In model selection, we prioritized AUC values and model interpretability. The SVM and LR models were selected for their high AUC stability and generalizability across datasets. These models also provided clearer interpretability, vital for deciphering prediction logic and aiding clinical decisions. Our LR model, with a nomogram and SHAP, offered a visual prognostic tool for acute cerebral infarction, achieving AUCs of 0.934 and 0.903 in training and validation sets, respectively. The SVM model excelled with AUCs of 0.935 and 0.916, underscoring its predictive strength in stroke prognosis. These outcomes affirm the models’ practicality in medical prognosis and set a basis for further analysis.

### 4.1 Logistic regression in medical applications

Logistic regression is a foundational classification algorithm in the machine learning repertoire, renowned for its capacity to map a linear combination of input features to a probability space via the Sigmoid function, which is instrumental in binary classification predictions (20). This model harnesses maximum likelihood estimation and gradient descent algorithms to optimize its parameters, effectively minimizing the log loss function. In the medical field, logistic regression plays a pivotal role in disease diagnosis, risk assessment, and prognosis judgment (21). It excels in the domain of disease prognosis by screening feature variables related to prognosis, constructing predictive models, and translating these models into nomograms—a visual tool that intuitively conveys the impact of different prognostic factors on outcomes, thereby providing statistical support for clinical decision-making.

Logistic regression, as a linear model, performs well in handling simple relationships but has significant limitations when dealing with complex nonlinear relationships. For example, it assumes a linear relationship between features and the target variable, which may prevent it from capturing complex interactions among features in clinical data (22). Additionally, logistic regression requires high linear independence among features; when multicollinearity is present, the stability and interpretability of the model can be compromised (23). In medical predictive modeling, these limitations of logistic regression may lead to insufficient predictive performance, especially in high-dimensional data environments.

Our model, echoing the robustness of other studies, demonstrates significant performance. It aligns with Zou’s research, where a logistic regression model predicted the prognosis of children post-transthoracic balloon pulmonary valvuloplasty with AUC values ranging from 0.723 to 0.870, indicating a reasonable predictive efficacy (24). In Wang’s study, a nomogram constructed using LASSO regression and logistic regression algorithms showcased high discriminative ability with AUC values up to 0.919 across multiple independent cohorts, demonstrating good calibration and clinical utility in predicting patients’ long-term postoperative recovery outcomes (25). Zhu’s prospective cohort study identified serum secretoneurin levels as an independent predictor of poor prognosis after intracerebral hemorrhage, with the developed multivariate logistic regression model and nomogram showing high accuracy in predicting poor outcomes for ICH patients after 90 days, with AUC values of 0.930 and 0.913, respectively (26).

The logistic regression model confirms its significant application in predicting cerebral infarction, paralleling the efficacy of models that achieved AUC values of 0.778 in training and 0.733 in validation for early ischemic neurological deterioration (27). In the challenging diagnosis and treatment of moyamoya disease, our model, akin to those in Sun’s study, excelled with AUC values as high as 0.891, 0.849, and 0.911 across different datasets, showcasing good calibration (28). Another study analyzed data from 243 moyamoya disease patients who underwent superficial temporal artery to middle cerebral artery (STA-MCA) bypass surgery, generating a nomogram through multivariate logistic regression analysis to predict good postoperative collateral circulation formation (PCF) after STA-MCA bypass surgery, with a concordance index (C index) of 0.88, exhibiting excellent calibration curves and good clinical application value (29).

Our model’s performance is commendable, offering insights and predictions that are on par with these precedents, further solidifying the utility of logistic regression in medical prognostication and decision support.

### 4.2 SVM in medical applications

Support Vector Machine (SVM) is a premier algorithm in the realm of supervised learning, celebrated for its prowess in identifying the most discriminative hyperplane that separates classes within high-dimensional spaces (30). Its strength in binary classification has positioned SVM as a go-to tool in medical applications, where it excels in image recognition, disease prediction, and bioinformatics due to its precision and robust generalization (31).

In the medical imaging domain, SVM has demonstrated its mettle by achieving a 67% accuracy rate in analyzing resting-state functional MRI data of patients with Tourette’s syndrome, with particular acumen in identifying key neural network traits (32). Its diagnostic utility extends to distinguishing Tourette’s Disorder patients from healthy controls based on brain functional connectivity and in assessing the impact of therapeutic interventions (33). In predictive analytics, SVM has been effectively leveraged to forecast the risk of mild cognitive impairment progressing to Alzheimer’s disease and to predict the recurrence of breast cancer post-treatment. Within bioinformatics, SVM has shown high accuracy and specificity in identifying NLRP3 inhibitors, underscoring its potential in drug discovery (34). In cerebrovascular disease research, SVM has been instrumental in identifying immune-related genes associated with ischemic stroke, marking a significant advancement in early diagnosis and treatment strategies (35).

Our implementation of the SVM model in predicting stroke patient outcomes has been nothing short of exemplary. The model achieved an impressive 89.5% accuracy on the training set, with an AUC of 0.9453, reflecting its strong predictive capabilities. On the test set, despite a modest dip to 79.4% accuracy, the AUC remained high at 0.9213, indicating the model’s ability to generalize well to new data. SHAP value analysis further validated the significance of hypertension and smoking history as pivotal risk factors, providing actionable insights that can inform clinical decision-making processes.

Of course, there are some limitations of the SVM, SVM is a powerful classification model, particularly effective in handling high-dimensional data, but its “black-box” nature limits model interpretability, which is a critical issue in medical applications. SVM has high requirements for data preprocessing, especially with low tolerance for outliers, which can compromise model robustness (36). Moreover, the training process of SVM is computationally intensive, especially with large-scale datasets, limiting its application in real-time prediction scenarios (37). In medical prediction, these limitations may affect the clinical applicability of the model, especially in scenarios requiring rapid decision-making.

### 4.3 Stroke risk factors and prognostic indicators

Diabetes is a significant independent risk factor for stroke, increasing the risk by 2–4 times and adversely affecting patient prognosis (38, 39). Proper diabetes management is crucial for reducing stroke incidence and improving outcomes. Our study supports the importance of active diabetes management in preventing ischemic stroke and enhancing patient prognosis.

Hypertension is a critical risk factor for stroke, with higher blood pressure levels increasing stroke risk and negatively impacting patient prognosis (40, 41). Antihypertensive treatment, especially when systolic pressure is over 140 mmHg, is effective in reducing stroke risk, likely due to its role in preventing vascular remodeling and atherosclerosis (42). Our study confirms that hypertensive stroke patients with poorly controlled blood pressure have worse outcomes, underscoring the need for active blood pressure management to decrease stroke risk.

Smoking is a significant independent risk factor for stroke, increasing the relative risk by 1.88 times compared to non-smokers, with risk escalating with smoking quantity (43). Quitting or reducing smoking significantly lowers stroke risk, and our study underscores the importance of smoking cessation for better stroke prognosis.

Platelet Distribution Width (PDW), a measure of the variability in platelet size within the bloodstream, has been implicated in the prognosis of cerebral infarction. Studies suggest a potential link between elevated PDW levels and adverse outcomes in patients with cerebral infarction. Shen’s research indicated that higher PDW levels correlate with poorer patient prognoses (44), possibly because increased PDW signifies heightened platelet metabolic activity, which could impede vascular recanalization and exacerbate post-stroke prognosis. A systematic review and meta-analysis further supports the predictive value of PDW and Mean Platelet Volume (MPV) in determining clinical outcomes for patients with acute ischemic stroke (2). These insights propose that PDW could be a valuable biomarker for the prognosis of cerebral infarction. Our study aligns with this notion, revealing that increased PDW levels are associated with severe stroke outcomes. In conclusion, PDW, as an indicator of platelet activity, may be correlated with the prognosis of cerebral infarction, although further research is needed to elucidate its precise mechanisms and clinical utility.

The National Institutes of Health Stroke Scale (NIHSS) score, Barthel Index (BI), and Watanabe Drinking Test (KWST) are essential tools for assessing stroke patients’ neurological function, daily living capabilities, and swallowing function, respectively. The NIHSS score is a key indicator of stroke severity, with higher scores reflecting greater neurological deficits and potentially poorer prognoses (45). The BI evaluates a patient’s capacity for activities of daily living, with lower scores indicating a poorer prognosis, higher mortality risk, and increased dependency in stroke patients (46). The KWST predicts stroke prognosis, closely with the severity of post-stroke dysphagia and well-correlated with both daily living activities and neurological deficits (47). Our study finds that patients with higher NIHSS scores, lower BI scores, and higher KWST scores are more likely to have worse outcomes. These assessments enable physicians to gain a comprehensive understanding of the conditions and prognoses of patients with cerebral infarction.

## 5 Limitation

Our study, while providing significant findings, has several limitations that warrant acknowledgment. First, the modest sample size and incomplete data for certain critical variables, such as C-reactive protein (C-RP) and B-type natriuretic peptide (BNP), may affect the robustness of our results. Second, despite an adequate model fit, the potential for multicollinearity among variables poses a risk of overfitting. Third, the study’s concentration on an Asian population restricts the generalizability of our findings to other ethnic groups. Additionally, the lack of multicenter external validation is a notable limitation, which we intend to address by broadening our participant base and integrating prospective data collection. Although the LR model demonstrated strong performance, the dip in accuracy and recall rate on the test set indicates a need for further enhancement in model generalization to new datasets. The reduced sensitivity on the test set may also suggest difficulties in identifying certain cases in practical applications. Future work will focus on refining the model and conducting more extensive studies to address these limitations and to strengthen the model’s predictive accuracy and clinical applicability.

## 6 Conclusion

We leveraged SHAP to elucidate the SVM model’s predictions and harnessed a nomogram to render the LR model’s outcomes more comprehensible. These machine learning methodologies, both interpretable and visually accessible, excelled in forecasting the prognostic risk factors for patients afflicted with acute cerebral infarction. Moreover, they offered substantial clinical utility by reinforcing medical decision-making and facilitating the communication of prognoses to patients, thereby bridging the gap between complex analytics and practical application in healthcare. We will refine these models for easier clinical application and validate their generalizability through extensive multicenter studies. We also aim to incorporate more interpretable ML techniques to improve model transparency and clinical relevance, offering tailored treatment strategies for stroke patients.

## Data Availability

The raw data supporting the conclusions of this article will be made available by the authors, without undue reservation.

## Supplementary material

SUPPLEMENTARY FIGURE 1 Univariate ROC Curve Area Under the Curve (AUC) Feature Ranking Plot **(A)** AUC Value Feature Ranking Plot **(B)**.

SUPPLEMENTARY FIGURE 2 Clinical Factors Correlation Heatmap.
