# Supplementary figures and images for: Interpretable prediction of stroke prognosis: SHAP for SVM and nomogram for logistic regression
Source: Front Neurol. 2025 Mar 4;16:1522868. doi: 10.3389/fneur.2025.1522868 (PMC11913711; doi:10.3389/fneur.2025.1522868)

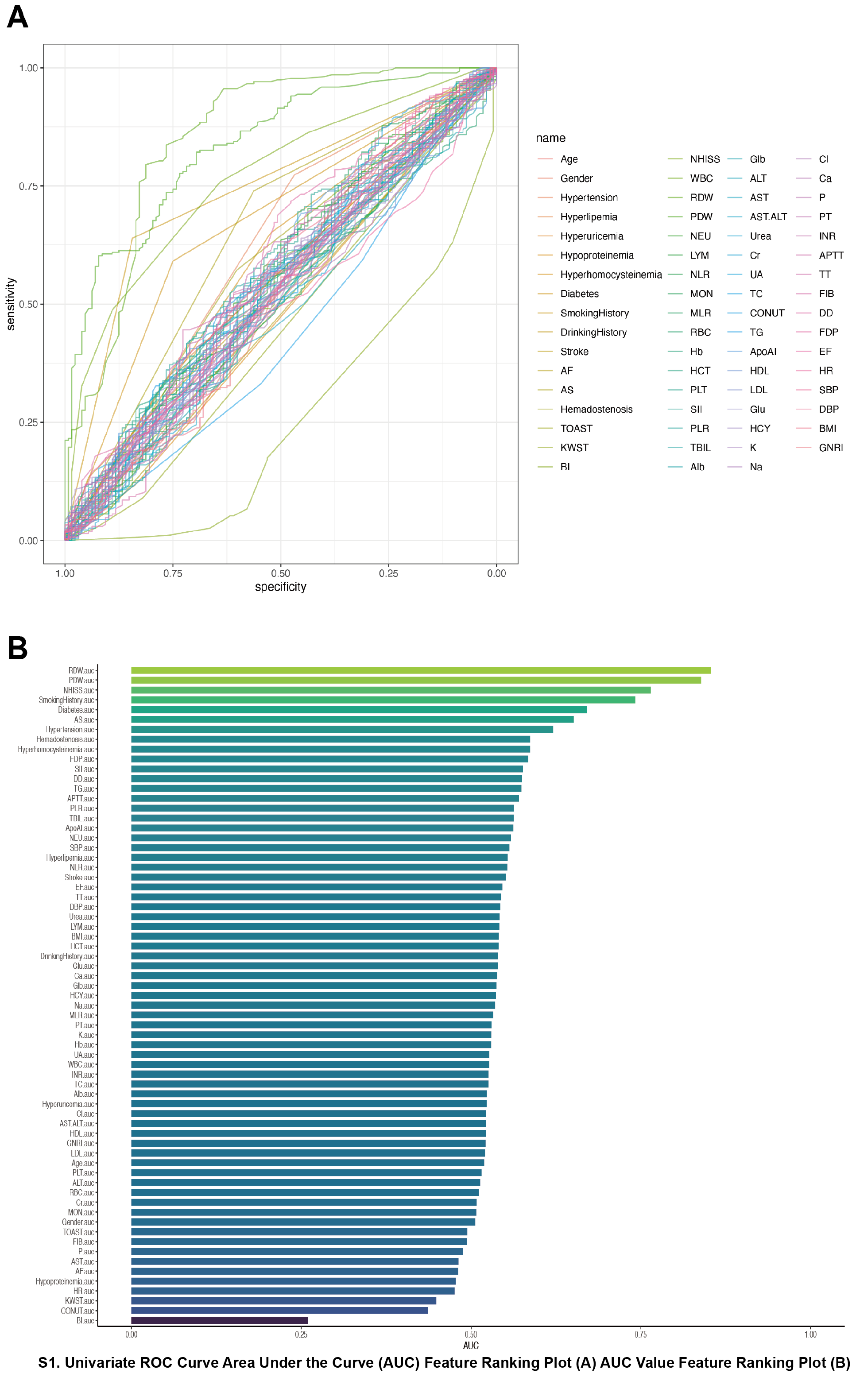

Supplement: SUPPLEMENTARY FIGURE 1 — Univariate ROC Curve Area Under the Curve (AUC) Feature Ranking Plot (A) AUC Value Feature Ranking Plot (B). [file Image_1.tif]

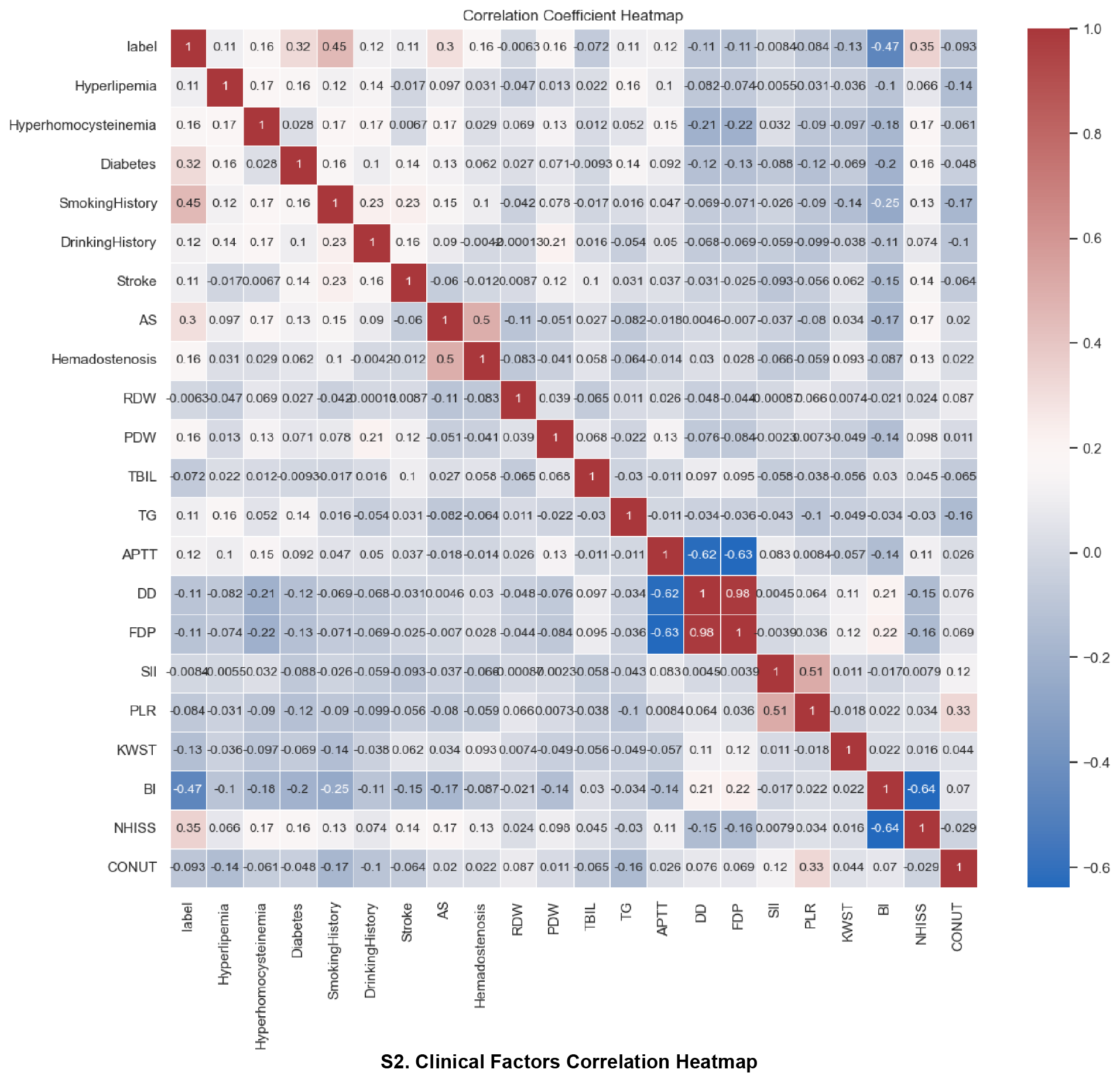

Supplement: SUPPLEMENTARY FIGURE 2 — Clinical Factors Correlation Heatmap. [file Image_2.tif]

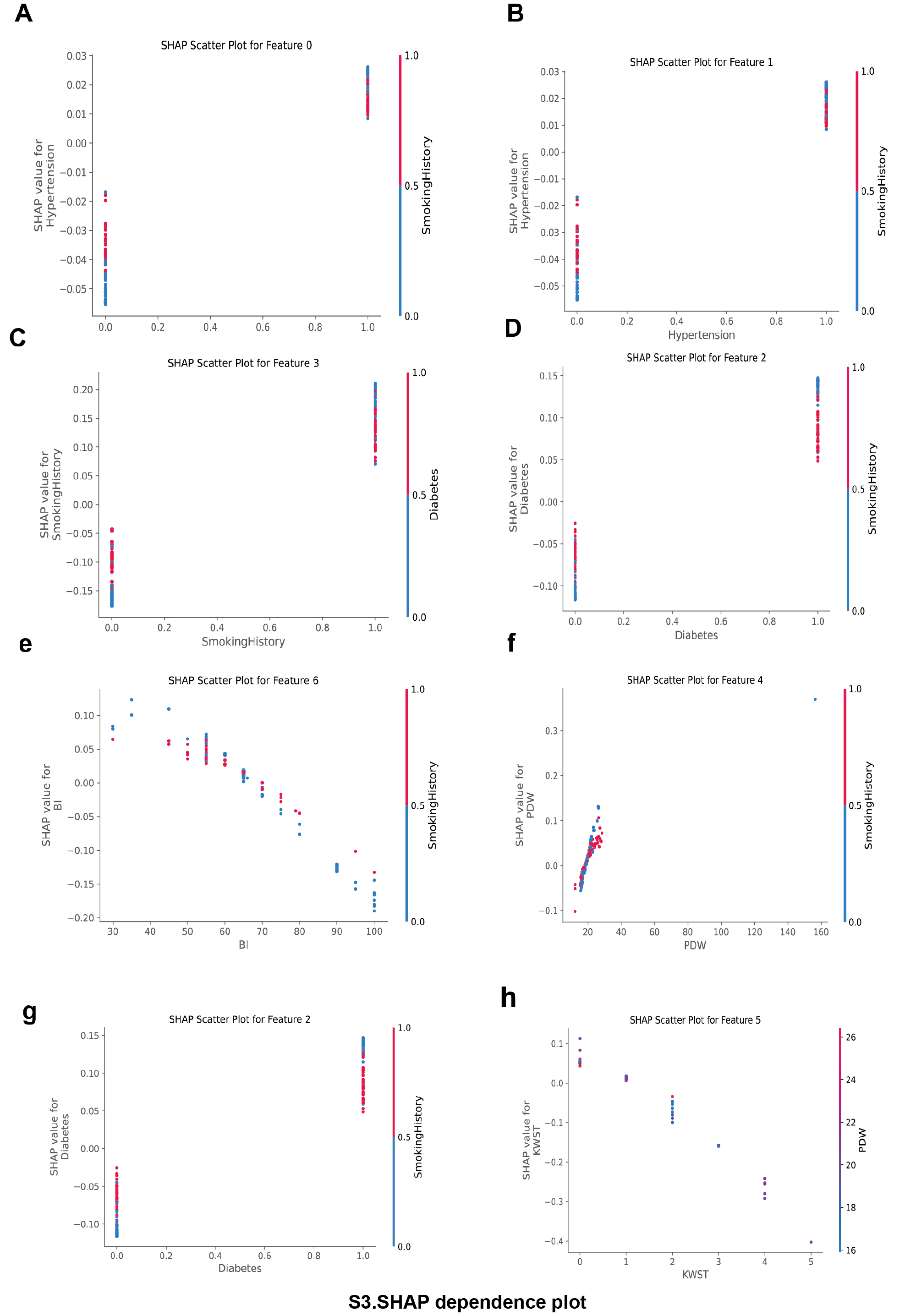

Supplement: Supplementary file 3 [file Image_3.tif]
